# A Case of Secondary Syphilis

**Published:** 1852-06

**Authors:** Robert Durrett

**Affiliations:** Louisville Marine Hospital


Art. III.—*A Case of Secondary Syphilis.* By Robert Durrett, M. D.

John Adams, about 17 years of age, a native of Kentucky, a mason by occupation, was admitted into the hospital March 23d, 1852. He states that his attention was attracted, about the middle of January last, "to a small yellow speck upon the prepuce." Two or three days afterwards the parts became so much swollen that it was impossible to retract the prepuce. Sanguineous purulent matter was discharged in considerable quantities from the part.

He entered the N. O. Hospital; received local treatment; remained 26 days; and was discharged cured as he thought about the 25th February.

Actual condition March 23d, 1852—Face, body and extremities literally covered with pustular eruption, with extensive ulceration about the external genital organs This eruption was discovered by the patient some twelve days previous to his admission into our Hospital.

*Treatment.*—Donovan's solution ter in die, and yellow wash to ulcers. This treatment was contined until the 3d of April, without any apparent benefit; but on the contrary new pustules appeared daily.

April 3d.—The patient is put upon protoiodid hydrarg.

April 4th.—The patient vomits frequently, and says his medicine makes him sick. He has erysipelas of the face, with considerable edema about the eyes. Ordered cold to face; a pill of blue mass, and Pulv. Dover, each grs. v. every four hours.

April 7th.—Patient's face is painted with tinct. iodine.

April 9th.—Patient relieved of erysipelas; discontinued blue mass; and resumed protoiod. hydrarg.

April 10th.—Genital organs very much enlarged by serous infiltration into cellular tissue, which is relieved by the application of acetate of lead and opium.

April 11th.—Patient mercurialized, pustular eruption disappearing.

April 13th.—Pustular eruption entirely disappeared, and the papular ensues over the entire body, resembling at a distacce *measles*. Stop mercury.

April 19th.—Papular eruption disappearing.

April 22d.—Papular eruptions entirely gone, leaving a squamous condition of the skin.

May 1st.—The patient is discharged cured, 38 days after he entered the Hospital, and near three months and a half after the appearance of syphilitic disease.

Not the least interesting feature in this case is the ensuing of the papulæ upon the disappearance of the pustular eruption, while the patient was under the specific effects of mercury. But in this, as in all cases, we should not stop short when surprised at the effects of medicine. The phenomena should be observed and the causes investigated. The appearance of this new eruption was not the effect of the remedy, but the gradual subsidence of the severe symptoms of the disease.

Louisville Marine Hospital, May 8th, 1852.

